# Transposon mutagenesis in bacterial natural product discovery

**DOI:** 10.1042/EBC20250023

**Published:** 2026-08-19

**Authors:** Mega F. Warsito, Napawit Nonthakaew, Marina Suppi, Liam K.R. Sharkey, Justin Nodwell, Sacha J. Pidot

**Affiliations:** 1 Department of Microbiology and Immunology at the Doherty Institute, University of Melbourne, Melbourne, 3000, Australia; 2 Department of Biochemistry, Temerty Faculty of Medicine, University of Toronto, Ontario M5G 1M1, Canada

**Keywords:** biotechnology, microbiology, transposons

## Abstract

Transposon mutagenesis has re-emerged as a powerful and versatile strategy for discovering and characterising specialised metabolites encoded by biosynthetic gene clusters (BGCs). While genomics has revealed an enormous diversity of putative BGCs across bacteria, many remain silent, weakly expressed, or genetically intractable, necessitating experimental tools that can link genotype to chemical output. Transposons provide an unbiased and broadly applicable platform for disrupting, activating, or modulating gene expression without relying on homologous recombination, making them particularly valuable in challenging microbial hosts. Here, we review the major applications of transposon mutagenesis in natural product discovery, providing examples that highlight discoveries made using phenotype- and bioactivity-guided screens, phenotype-independent strategies, and transposon-based engineering of heterologous expression platforms. Transposon technologies provide flexible and scalable tools for activating, characterising, and engineering microbial BGCs. As genome mining continues to unearth rich seams of unexplored metabolic potential, these tools will remain essential for converting genetic predictions into chemical discovery.

## Introduction

Natural products (NPs), also known as secondary or specialised metabolites, are structurally diverse small molecules produced by bacteria, fungi, plants, and invertebrates [1]. Unlike primary metabolites, which are essential for growth and reproduction, NPs are non-essential for growth but play important ecological roles mediating interactions among microbes, plants, and animals, including competition, symbiosis, and defence [2,3]. In addition, NPs have contributed disproportionately to the discovery of new antibiotics, antifungals, anti-cancer agents, immunosuppressants, and agrichemicals [4].

The production of NPs is encoded within biosynthetic gene clusters (BGCs), co-localised sets of genes that encode the enzymes, regulators, self-resistance, and transporters required to produce a given NP [5]. BGCs range in size from 10 kb up to >150 kb of DNA and can encode a broad range of biosynthetic logics from the large modular assembly lines of polyketide synthases (PKS) and nonribosomal peptide synthetases (NRPS) to the enzymatically minimalist ribosomally synthesised and post-translationally modified peptides (RiPPs), as well as hybrid systems that blur classical biosynthetic boundaries [6]. This genetic organisation underlies the combinatorial richness of NP scaffolds, allowing extraordinary structural diversity to be produced from simple precursors.

The development of computational tools for identifying BGCs within genomes has massively expanded our view of BGC diversity and accelerated the discovery of novel NPs [7]. While culture-based NP discovery has revealed a wealth of molecules primarily from actinomycetes, genome mining has revealed that many microbes encode far more BGCs than compounds they produce under laboratory conditions, especially in underinvestigated groups such as anaerobes, myxobacteria, cyanobacteria, *Burkholderia*, and Pseudomonads [8,9]. This discrepancy highlights the prevalence of silent or cryptic BGCs, which remain transcriptionally inactive or expressed at very low levels in standard laboratory conditions [10]. Unlocking these silent clusters is one of the major challenges in contemporary NP discovery and has motivated the development of genetic, chemical, and cultivation-based strategies for BGC activation.

Genetic manipulation is a key technique for both linking BGCs to their cognate products and activating silent BGCs. Traditional gene knockout methods have enabled targeted interrogation of individual biosynthetic steps, but these methods are often slow, species-specific, or limited by low transformation efficiencies. More recently, genome engineering technologies, such as CRISPR-Cas systems, recombineering, and promoter engineering, have accelerated BGC functional analysis and heterologous expression [11–13]. However, many microbial producers remain genetically intractable or difficult to manipulate (such as those in the genera *Micromonospora*, *Actinomadura*, and *Saccharothrix*). Introducing targeted edits can be difficult in strains with robust restriction–modification systems or complex life cycles (such as *Myxococcus*). For these organisms, unbiased or semi-random mutagenesis approaches remain indispensable.

**Figure 1:**
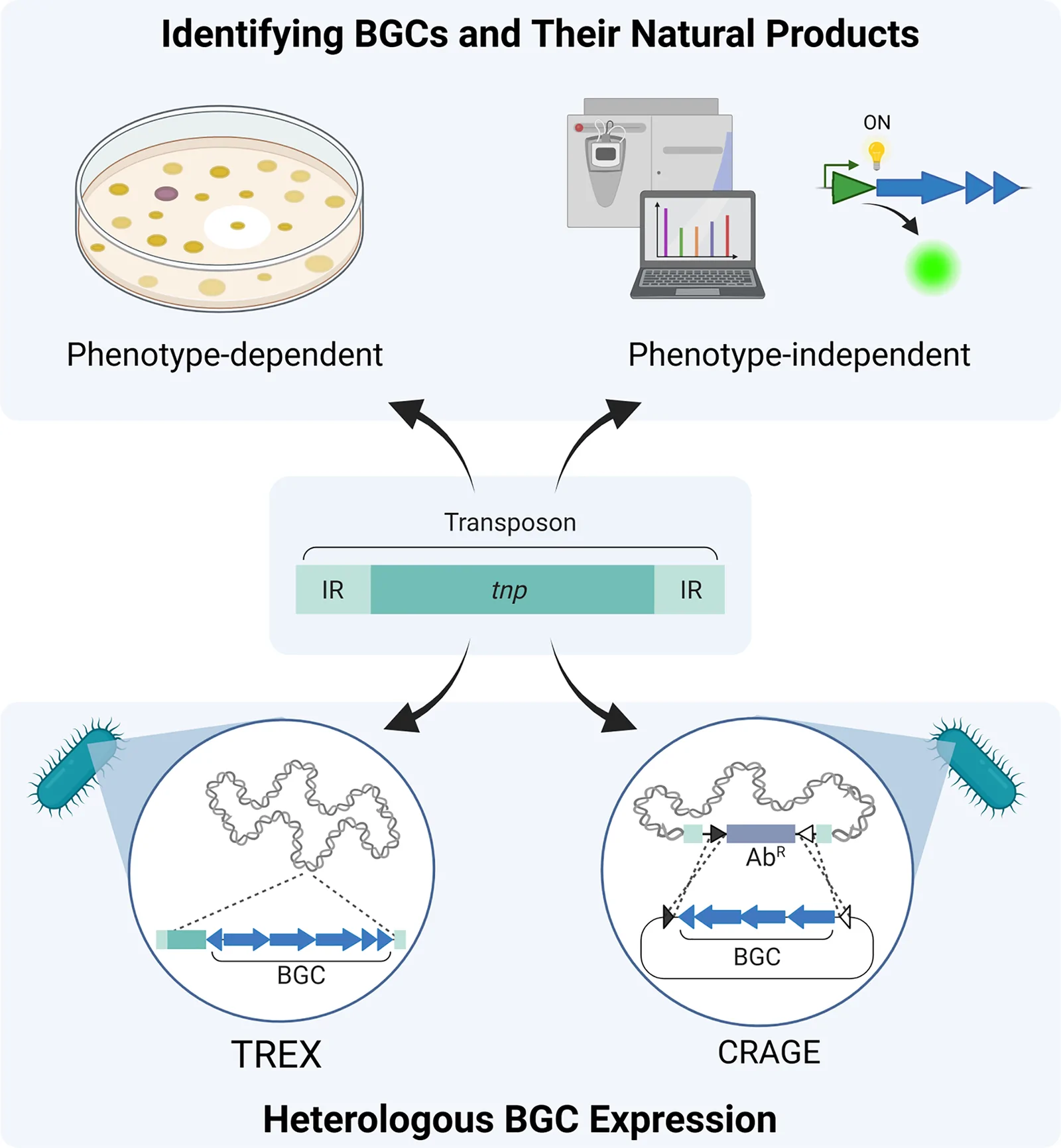
Summary of ways in which transposons have been used in NP discovery Phenotype-dependent screens rely on altered properties of the transposon mutants, such as altered colony morphology or modification of antibiotic-producing phenotype. Phenotype-independent approaches rely on direct measurement of new molecules or tracking via production of fluorescent proteins. Heterologous BGC expression has been simplified using systems such as TREX and CRAGE, which use transposons as carriers of BGCs for the purposes of integration into the genome of a heterologous host. For further details of each method, refer to the appropriate section in the text. IR, inverted repeat; *tnp*, transposase-encoding gene; BGC, biosynthetic gene cluster; TREX, transfer and expression of biosynthetic pathways; CRAGE, chassis-independent recombinase-assisted genome engineering.

Among these tools, transposon mutagenesis has emerged as a versatile and broadly applicable method for investigating secondary metabolism [10], and the main ways in which transposons are applied to NP discovery are summarised in Figure 1. Transposons are mobile genetic elements that move from one DNA site to another by a process called transposition [14]. Most transposons used for mutagenesis transpose by a ‘cut-and-paste’ mechanism: a dedicated transposase binds the terminal inverted repeats of the transposon, excises it from the donor DNA, and integrates it into a new target site, typically generating a short target site duplication at the insertion site [14]. Different transposon families show characteristic target site preferences; for example, mariner-family transposons such as *Himar1* recognise the simple dinucleotide sequence TA, whereas the Tn*5* transposon does not have a strict consensus sequence, rather targeting GC-rich DNA sequences [15,16]. Because transposition does not require homologous recombination, these systems operate effectively in many organisms that are otherwise genetically recalcitrant.

These mechanistic properties can be used to construct distinct types of transposon libraries [14–16]. For example, in a random insertion library, a transposon such as Tn*5* is delivered so that each mutant carries a single insertion at an essentially unpredictable genomic position; a ‘Tn*5* transposon library’ therefore refers to a population of mutants created using a Tn*5*-based transposon to generate broad, quasi-random genome coverage. In contrast, a *Himar1* transposon mutant library is constructed using a mariner-family *Himar1* element, which inserts specifically at TA dinucleotides. Both types of transposons allow construction of saturating libraries, disrupting most non-essential genes and enabling high-resolution mapping of genotype-phenotype relationships (Table 1) [15]. Within the context of secondary metabolism, insertions that disrupt genes directly within a BGC and abolish compound production can rapidly link biosynthetic pathways to products.

**Table 1:** Comparison of the two major types of transposon systems (Tn*5* and *Himar1*) used in NP discovery

Transposon type	Mechanism	Target site preference	Typical delivery methods	Typical applications
Tn*5* (and engineered variants)	Cut-and-paste transposition	Relatively broad target range; preference for GC-rich regions as targets	*In vitro* transposition reactions; electroporation of pre-tagged DNA, conjugative delivery of ‘mini-Tn’ containing plasmids	Random insertion libraries, general mutagenesis
*Himar1* (and mariner family transposons; hyperactive variants)	Cut-and-paste; single-component transposase	Inserts at TA dinucleotides (high genomic coverage in many species)	Delivery via suicide or conjugative vectors, transposase expressed in trans	Saturating insertion libraries, Tn-seq, functional genomics in diverse hosts

Transposons can also activate silent BGCs when they insert near promoters or disrupt repressors, revealing metabolites that would otherwise remain undetected [10]. Furthermore, transposons engineered to deliver expression cassettes, fluorescent reporters, or strong promoters into genomes can be used for reporter-guided screening, transcriptional activation, and heterologous expression [10]. Collectively, these approaches allow researchers to connect genotype to phenotype, discover new molecules, and dissect the complex regulatory circuits that govern secondary metabolism.

**Figure 2:**
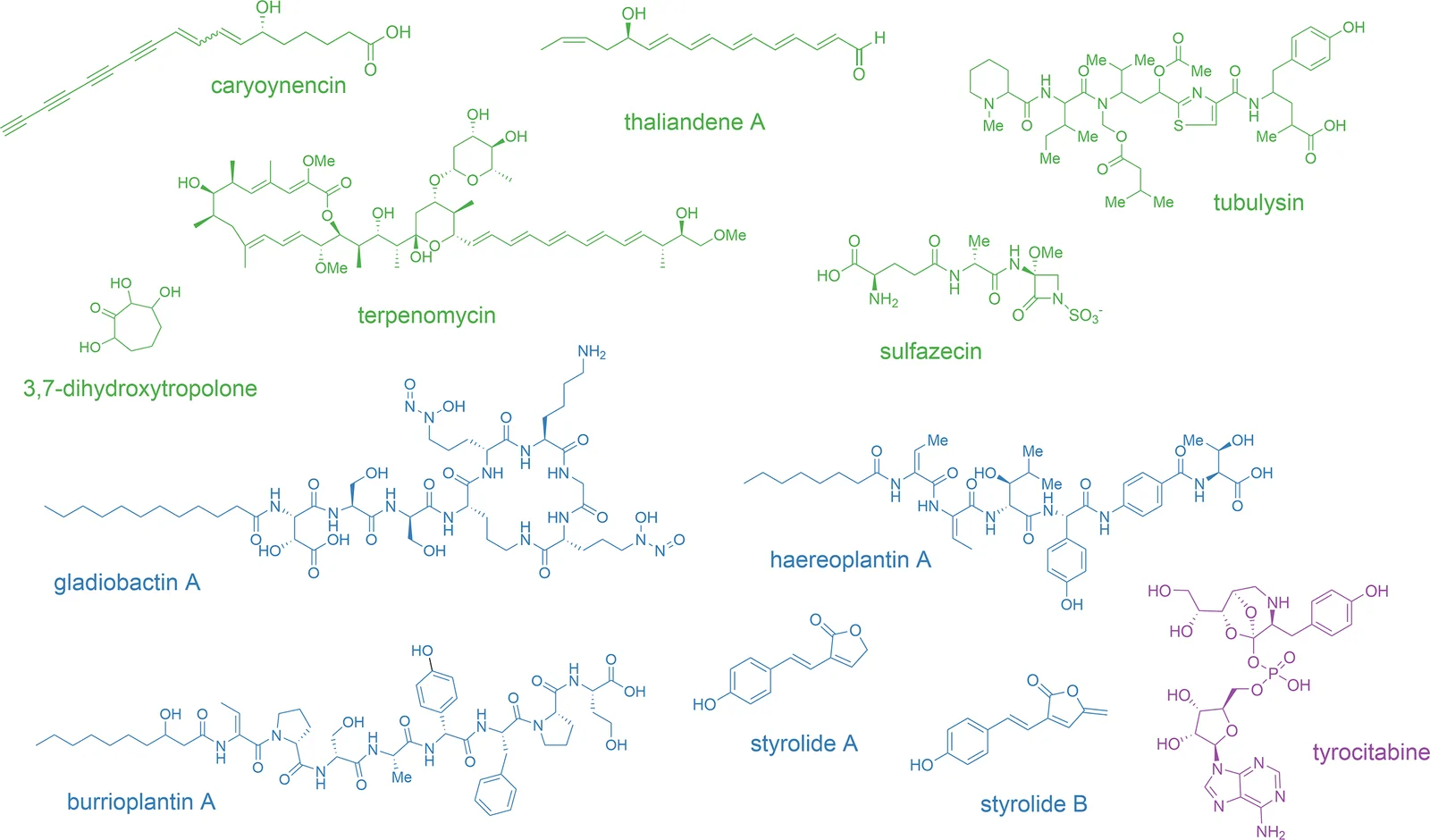
New NPs identified through transposon mutagenesis or transposon-based heterologous expression of their corresponding BGCs Molecules in green were identified by phenotype-dependent approaches, molecules in blue were identified by phenotype-independent approaches, and molecules in purple were identified by heterologous expression.

As interest in genome-guided NP discovery continues to grow, transposons occupy a unique position at the intersection of classical microbial genetics and modern synthetic biology. Their simplicity, broad host range, and adaptability make them an enduring and powerful tool for probing the function, regulation, and expression of BGCs. Here, we review the different uses of transposons as tools to identify novel NPs and their biosynthetic pathways in a range of bacteria. An overview of these approaches is provided in Figure 1, and the structures of new NPs whose BGCs were identified or expressed using these methods are shown in Figure 2.

## Using transposons to identify BGCs and their products

As a powerful tool to connect genotype to phenotype, transposon mutagenesis has played an important role in identifying biosynthetic pathways, revealing regulatory architecture, and uncovering novel specialised metabolites. Broadly, the use of transposons for BGC discovery can be organised into two major conceptual approaches: (i) phenotype- or bioactivity-guided screens, where the loss or alteration of a biological trait highlights mutants with altered metabolite production; and (ii) phenotype-independent or chemically guided approaches, where metabolite profiling, transcriptomics, or reporter activation reveals BGCs regardless of their visible or biological effects.

### Phenotype or bioactivity-guided approaches

The earliest and most intuitive application of transposon mutagenesis for BGC discovery relies on screening large mutant libraries for altered biological activity or visible phenotypes. Loss of antimicrobial activity, changes in pigmentation, or disruption of developmental processes can provide rapid readouts of biosynthetic gene disruption, even when the underlying chemistry is unknown.

Loss of bioactivity from an antibiotic-producing strain is a simple readout that can be rapidly applied to screen transposon libraries. An example of this is the identification of the biosynthetic pathway for the polyyne caryoynencin from *Burkholderia caryophylli* DSM 50341 (Figure 2) [17]. Although the compound had been known since 1987 [18], its biosynthetic basis was only identified when a *Himar1*-based transposon library in the producing strain was screened for mutants lacking antibacterial activity or pigment production. Mapping the insertion sites revealed disruption of a desaturase gene essential for polyyne formation, leading to previously unknown genes for caryoynencin and the first description of a bacterial polyyne pathway [17]. A similar approach was taken in *Pseudomonas acidophila* ATCC 31363 to identify the BGC for sulfazecin A (Figure 2). Here, a Tn*5* library in *P. acidophila* was screened against a β-lactam-sensitive *Escherichia coli* strain, identifying a *P. acidophila* mutant that had lost anti-*E. coli* activity [19]. Identification of the insertion site showed that it had disrupted an NRPS, allowing direct assignment of the sulfazecin BGC [19]. Likewise, a *Himar1* library in the plant-derived *Pseudomonas* sp. PS652 identified a BGC responsible for its activity against the potato pathogen *Streptomyces scabiei* [20]. This BGC, previously undetected *in silico* by genome mining tools, was later shown to encode the production of 3,7-dihydroxytropolone (Figure 2), demonstrating how transposon mutagenesis can reveal cryptic BGCs not predicted computationally [20].

Bioactivity-guided approaches are not restricted to antibacterial activity, as shown by the identification of the biosynthetic pathway for the cytotoxin tubulysin (Figure 2). Tubulysin, produced by the myxobacterium *Angiococcus disciformis* strain An d48, was first identified at the start of the current millennium [21] and has generated significant interest as a candidate for use in anti-cancer antibody–drug conjugates [21,22]. However, as no [23] genetic systems were available to manipulate the producing strain, the biosynthetic locus remained enigmatic until a *Himar1* transposon mutant library was generated in the producing strain [24]. From only 1200 mutants, the authors used a microscopy-based cell nucleation assay to identify four mutants that did not produce tubulysin, with all mutants having insertions within the same BGC, identifying it as the source of tubulysin [24].

Altered colony phenotypes and morphologies can also reveal new NPs. As an example, transposon mutagenesis in *Burkholderia thailandensis* strain DW503 using a mini-Tn*5* cassette revealed five orange colonies (wild-type *B. thailandensis* colonies are white), suggesting the production of a new pigment [25]. Subsequent metabolomic analyses revealed the pigment was related to the production of previously uncharacterised polyene thailandenes (Figure 2) [25]. Mapping of the transposon insertion site suggested that it had induced a previously silent type I PKS-encoding gene, possibly through polar effects of the insertion [25]. One of these orange-pigmented mutants was selected for a second round of transposon mutagenesis to identify the thailandene pathway, which proved to be a three-gene operon encoding a type I PKS with a non-canonical domain arrangement [25].

In a similar fashion, Xu et al. built on previous work using a *Streptomyces* codon-optimised and hypermutable Tn*5* [26,27] to generate a large (>50,000 mutant) transposon library in *Streptomyces coelicolor* M145. With such a large library, the authors investigated genes that influenced the production of the two commonly produced *S. coelicolor* pigments: the red-coloured secondary metabolite undecylprodigiosin [28] and the blue-coloured compound actinorhodin [29]. Both studies identified hundreds of insertions outside of the undecylprodigiosin and actinorhodin BGCs that influenced the production of these metabolites. Further analysis of the inactivated genes revealed that genetic regulators were overrepresented among the genes both positively and negatively influencing actinorhodin and undecylprodigiosin production [28,29]. Further work on several of these mutants identified a regulator, OrrA, whose target is *wblA*, encoding a pleiotropic regulator of secondary metabolism, and revealing a complex and previously unrecognised regulatory cascade [30]. Other groups have further utilised the same Tn*5* system to reveal regulators for the production of the antifungal candicidin [31]. The same approach has also been applied in rare and intractable Actinobacteria, such as *Nocardia terpenica* AUSMDU00012715, to identify the genetic basis for the production of the novel cytotoxin terpenomycin (Figure 2) [32].

Taken together, these examples illustrate how transposon mutagenesis, in combination with phenotype-based screening strategies, can be used to uncover biosynthetic pathways and new NPs.

### Phenotype-independent approaches

While phenotype-based screens are powerful, many BGCs do not produce compounds with obvious biological activity or visible phenotypic changes. In these cases, phenotype-independent strategies are essential and generally seek to utilise reporter genes or direct metabolite detection methods to screen a library of transposon mutants. Such approaches can reveal new metabolites and their biosynthetic pathways when no overt phenotype is present, as outlined in the examples below.

The use of fluorescent reporters to identify BGCs that have been activated following transposon mutagenesis represents a novel phenotype-independent strategy. Building upon previous reporter-guided approaches [33], Mao et al. integrated a range of fluorescent reporters within several silent BGCs in *B. thailandensis* strain E264 [34]. Subsequent transposon mutagenesis using a modified T8 transposon allowed high-throughput screening for mutants with elevated fluorescence, serving as a proxy for pathway activation [34]. One identified mutant showed a dramatic 26-fold increase in malleilactone production, which was linked to a transposon insertion in *pyrF*, highlighting a link between secondary metabolism and pyrimidine biosynthesis [34]. Further work confirmed this link by chemical inhibition of PyrF using 5-fluoroorotic acid [34]. Although powerful, such reporter-based approaches require prior genetic manipulation to install reporter constructs, limiting their use to strains that are sufficiently tractable.

A more direct, phenotype-independent readout of specialised metabolism can be seen in the combination of transposon mutagenesis and screening for metabolite production. Again, using *Burkholderia*, Yoshimura et al. identified the novel metabolites haereoplantin and burrioplantin from *Burkholderia plantarii* ATCC 43733 and gladiobactin from *Burkholderia gladioli* ATCC 10248 by utilising LC-MS and imaging mass spectrometry (IMS) of Tn*5* mutant libraries (Figure 2) [35]. In this case, insertions in a putative DNA/RNA helicase and in *potF*, encoding a putrescine transporter, were associated with inducing haereoplantin/burrioplantin and gladiobactin production, respectively [35]. A similar approach using LC-MS-based screening of 2000 *mariner* mutants in *Pseudomonas entomophila* DSM 28517 revealed a cryptic two-gene biosynthetic pathway for the biosynthesis of labradorin that is also widespread in *Burkholderia* species [36]. In an even simpler approach, the biosynthetic pathway to the styrolides (Figure 2), butenolides potentially involved in bacterial signalling, was identified in *Pseudomonas fluorescens* HKI0874 [37]. This was achieved by thin-layer chromatography analysis of extracts from 2300 Tn*5* mutants [37]. An important point to note is that in these studies, BGCs and new secondary metabolites were identified from a relatively small number of mutants, meaning that even non-saturating transposon libraries are sufficient to identify new metabolites and their corresponding biosynthetic pathways.

Phenotype-independent approaches can also be used in strain engineering efforts to improve the production of known secondary metabolites. For example, Luo et al. [38] employed a reporter-guided Tn screening strategy to enhance production of the lipopeptide antibiotic daptomycin in *Streptomyces roseosporus* L30. They constructed a reporter-guided screening system by integrating a neomycin reporter cassette fused to the promoter of the daptomycin BGC, *dptEp*, into the chromosome [39]. Subsequent *Himar1*-based random mutagenesis and selection for mutants exhibiting high kanamycin resistance led to the identification of PhaR, a transcriptional regulator that negatively controls expression of the daptomycin BGC [38]. Remarkably, the *phaR* mutant exhibited approximately a 6.14-fold increase in daptomycin yield compared with the wild-type strain. This example demonstrates how Tn-based regulatory screening can be exploited to identify genetic targets for metabolic pathway up-regulation and strain improvement.

Taken together, these examples highlight how chemical-first approaches can reveal BGCs even when neither genome mining nor conventional phenotypic screens provide clear leads and can help untangle the often complex regulatory pathways that govern the production of secondary metabolites.

## Using transposons to transplant BGCs

Heterologous expression remains one of the most powerful strategies for accessing the products of BGCs, particularly when native hosts are slow-growing, genetically intractable, or contain regulatory barriers that suppress metabolite production [40]. However, the routine introduction of large BGCs into surrogate hosts is still technically challenging, as traditional chromosomal integration methods often require multiple cloning steps or low-efficiency recombination events, and many BGCs exceed the size limits of standard plasmid systems.

Transposons offer an attractive alternative because they can deliver genetic cargo to a variety of genomic locations without the need for a specific integration site. This has enabled the development of ‘landing-pad’ systems: engineered chromosomal sites that serve as universal integration points for large DNA fragments, including complete BGCs (Figure 1) [41]. In general, these systems operate through a two-step process: (i) a transposon installs a recombination-competent landing pad at a random genomic location; and (ii) the BGC of interest, carried on a second construct and flanked by matching recombination sites, is integrated precisely and efficiently into the landing pad by a dedicated recombinase. This modular framework improves integration efficiency, supports multi-copy or multi-locus integration strategies, and allows BGCs to be expressed under well-defined promoters in genetically stable chromosomal contexts [41].

Several major systems have been developed using this principle, each suited to different host ranges or experimental goals. The first of these to be developed was chassis-independent recombinase-assisted genome engineering (CRAGE) [42] (illustrated in Figure 1), which is based on recombinase-assisted genome engineering (RAGE) developed in *E. coli* [43]. CRAGE was developed to extend landing pad-based BGC expression to be host-independent, allowing access to strains that are difficult to cultivate or genetically manipulate. In its original iteration, CRAGE used a *mariner* transposon that inserts a standardized landing pad containing a selectable marker and the Cre-recombinase flanked by *lox* sites [42]. The construct also includes T7 RNA polymerase under control of the *lacUV5* promoter [42]. Once the landing pad is integrated, a second plasmid carrying the BGC under the control of the T7 promoter and flanked by *lox* sites is introduced, allowing precise integration of the BGC into the landing pad and excision of the original antibiotic marker and Cre recombinase gene [42]. CRAGE was originally used to test the expression of nine *Photorhabdus luminescens* BGCs in 23 different γ-proteobacteria from 11 different genera [42]. While not effective for every BGC and host combination, the system was highly versatile with known products from known BGCs expressed in a wide variety of hosts, and five new compounds were discovered from cryptic BGCs [42].

Further work has taken the CRAGE approach and applied subtle variations to express BGCs across a range of hosts. For example, Ke et al. sought to identify improved hosts for the production of phenazines, a group of NPs with diverse bioactivities [44]. By introducing phenazine BGCs into the same 23 proteobacteria, several strains were identified that were capable of producing phenazine precursors at hundreds of milligrams per litre [44]. A challenging aspect of the present study was the use of refactored phenazine BGCs derived directly from Actinobacteria, which have significantly different codon usage than the host proteobacteria [44]. Other authors have used CRAGE to express bioactive metabolites, such as naringenin in *E. coli* isolated directly from the human gut, as well as probiotic strains [45]. CRAGE has also been used to transfer BGCs from genetically intractable Clostridia into the more readily modified anaerobic acetogen *Eubacterium limosum*, identifying a clostridial pathway to phevalin production [46]. In addition, other studies have also taken the CRAGE approach and redesigned BGCs for integration and expression in both prokaryotes and eukaryotes, leading to the identification of the unusual nucleotide metabolite tyrocitabine (Figure 2) [47]. Lastly, the original CRAGE system has been updated to use sequential rounds of Cre/*lox* recombination to incorporate either Cas9 for gene deletion or a dCas9-RNAPω fusion for gene activation [48]. This provides a method to delete or activate genes within BGCs already in the host genome, avoiding complex cloning steps that are required with the original CRAGE protocol [48].

While all CRAGE systems discussed so far have used *mariner* to integrate the landing pad into proteobacterial genomes, an alternate approach has been applied in the Actinobacteria. Similar to CRAGE, the doubly transposition and site-specific recombination (dTSR) approach was designed to achieve chromosomal integration of large BGCs in actinomycete genomes [49]. The system uses modified mini-Tn*5* transposons containing a phage integrase attachment site (*attB*) to insert the sites in the chromosome at a random location. A BGC of interest, carried on a separate bacterial artificial chromosome containing *attP* and the corresponding phage integrase, is then integrated at the inserted *attB* site [49]. To enhance expression, the landing-pad transposon used in the first integration round carries accessory genes. Most notably, it includes* bldA*, which enables translation of rare TTA codons commonly found in actinomycete regulatory genes, and *sfp*, encoding a promiscuous phosphopantetheinyl transferase necessary for activating many PKS and NRPS enzymes. The system has also been designed for multi-copy integration, with subsequent steps introducing a second transposon carrying an additional *attB* site at a separate genomic location, allowing a second copy of the BGC to be integrated [49]. This iterative strategy was used to significantly improve production of the insecticide spinosad in *Saccharopolyspora erythraea* LJ161 [49]. Cells containing one copy of the spinosad BGC produced 5.6–30.5 mg/l of the compound, while two copies increased titres roughly 4.5-fold to 137 mg/l, highlighting the utility of multi-copy chromosomal engineering for strain improvement [49].

Another system based on the transposon Tn*5* is the ‘transfer and expression’ (TREX) system, which combines Tn*5* transposition with two outward-facing T7 promoters to support heterologous expression of BGCs in diverse Gram-negative hosts (Figure 1) [50]. Here, instead of a landing pad being first integrated into the genome, the BGC of interest is cloned and surrounded by two complementary cassettes, each containing the Tn*5* mosaic ends (also known as inverted repeats). Following conjugation into a heterologous host, expression of the Tn*5* transposase (contained on one of the cassettes) allows integration of the entire BGC into the host genome at a random site [50]. Each cassette also includes an outward-facing T7 promoter, allowing functional expression of BGC genes cloned in the correct orientation. TREX has been used to functionally express the BGCs for zeaxanthin and prodigiosin from *Pantoea ananatis* in multiple hosts, including *Pseudomonas putida* KT2440, *E. coli* BL21 (DE3), and *Rhodobacter capsulatus* B10S, demonstrating its robustness across species with diverse metabolic capacities [50]. To further improve upon the time-consuming cloning step involved in TREX, the authors also developed yTREX, which uses yeast homologous recombination to assemble BGCs rapidly and accurately in a single step [51]. The resulting constructs can be transferred directly into the TREX framework for chromosomal insertion into bacterial hosts. Using yTREX, six BGCs encoding pigments such as prodiginines, violacein, and phenazines were cloned and integrated into *Pseudomonas putida* KT2440, enabling production of multiple structurally diverse metabolites from a single platform host [52].

Overall, these transposon-based systems provide powerful tools for activating silent BGCs, characterising pathways that are difficult to study in their native hosts, and enabling scalable engineering of production strains. Although each system differs in host range, integration strategy, and regulatory architecture, all leverage the ability of transposons to integrate DNA, providing tailored sites for stable chromosomal integration of large biosynthetic pathways.

## Conclusions and future directions

Transposon mutagenesis has evolved from a classical genetic tool into a versatile, multi-purpose platform that continues to reshape NP discovery. By enabling unbiased, high-throughput disruption or activation of genes, transposons provide a direct and efficient route to linking BGCs with their metabolic outputs. Phenotype- and bioactivity-guided screens remain powerful for identifying pathways underlying antimicrobial activity, pigmentation, and other easily measurable traits, whereas phenotype-independent strategies broaden this capability to include silent, cryptic, or weakly expressed BGCs through reporter activation or metabolomics-driven discovery. Together, these complementary approaches have illuminated previously unrecognised biosynthetic pathways, uncovered novel regulatory networks, and expanded the catalogue of known specialised metabolites across diverse bacterial taxa.

Beyond pathway discovery, transposons now play a central role in enabling heterologous expression of large and complex BGCs. Modern landing pad systems harness transposition to simplify chromosomal integration, refactoring, and multi-copy installation of biosynthetic loci in genetically tractable hosts. These technologies overcome long-standing barriers posed by native producers and support scalable, chassis-independent exploration of NP diversity.

Future work is likely to further expand the scope of transposon-based approaches for BGC discovery, characterisation, and exploitation. For example, high-density, barcoded transposon libraries coupled with condition-specific screening and metabolomics may enable systematic mapping of BGC function and regulation across diverse ecological niches, including host-associated and community contexts. In addition, further advances in transposon-assisted capture and transfer of large BGCs, together with increasingly sophisticated landing pad frameworks (CRAGE-like systems in multiple chassis and genomic loci), will streamline heterologous expression and multi-copy pathway installation. Finally, engineering transposases with altered target specificities or broadened host ranges may provide a way to extend these strategies to currently inaccessible taxa, thereby helping to convert the rapidly growing catalogue of predicted BGCs into experimentally validated chemistry.

As genome mining continues to reveal vast reservoirs of uncharacterised BGCs, transposon-based methods will remain essential for converting genetic potential into chemical reality, accelerating both discovery and engineering of microbial NPs.

## Summary

- Transposon mutagenesis provides an unbiased, broadly applicable tool for linking BGCs to their specialised metabolites.
- Phenotype- and bioactivity-guided screens can be used to rapidly identify disrupted BGCs, revealing both known and previously cryptic pathways.
- Phenotype-independent screening approaches can uncover induced BGCs, enabling discovery in cases where no overt biological phenotype exists.
- Transposon-based engineering platforms enable efficient, stable chromosomal integration and heterologous expression of large BGCs, overcoming historical limitations in refactoring and expressing pathways from intractable hosts.
- Transposon technologies provide flexible, scalable strategies for activating, characterising, and engineering microbial BGCs, accelerating NP discovery and expanding access to novel specialised metabolites.

## Open Access

Open access for this article was enabled by the participation of the University of Melbourne in an all-inclusive Read & Publish agreement with Portland Press and the Biochemical Society under an agreement with CAUL.

## Abbreviations

BGCs: biosynthetic gene clusters
CRAGE: chassis-independent recombinase-assisted genome engineering
CRISPR: clustered regularly interspaced short palindromic repeats
dTSR: doubly transposition and site-specific recombination
IMS: imaging mass spectrometry
LC-MS: liquid chromatography-mass spectrometry
NPs: natural products
NRPS: nonribosomal peptide synthetase
PKS: polyketide synthase
RAGE: recombinase-assisted genome engineering
RiPPs: ribosomally synthesised and post-translationally modified peptides
TREX: transfer and expression system
yTREX: yeast-based transfer and expression system
